# Antioxidative potential and ameliorative effects of green lentil (*Lens culinaris* M.) sprouts against CCl_4_-induced oxidative stress in rats

**DOI:** 10.3389/fnut.2022.1029793

**Published:** 2022-11-11

**Authors:** Hassan Barakat, Saleh I. Alshimali, Abdulkarim S. Almutairi, Raghad I. Alkhurayji, Sarah M. Almutiri, Thamer Aljutaily, Reham M. Algheshairy, Raghad M. Alhomaid, Rashed A. Aljalis, Mohammed F. Alkhidhr, Ahmed A. H. Abdellatif

**Affiliations:** ^1^Department of Food Science and Human Nutrition, College of Agriculture and Veterinary Medicine, Qassim University, Buraydah, Saudi Arabia; ^2^Food Technology Department, Faculty of Agriculture, Benha University, Banha, Egypt; ^3^Department of Veterinary Medicine, College of Agriculture and Veterinary Medicine, Qassim University, Buraydah, Saudi Arabia; ^4^Department of Pharmaceutics, College of Pharmacy, Qassim University, Buraydah, Saudi Arabia; ^5^Department of Pharmaceutics and Pharmaceutical Technology, Faculty of Pharmacy, Al-Azhar University, Assiut, Egypt

**Keywords:** *Lens culinaris*, sprouts, antioxidative potential, hepatoprotection, nephroprotection

## Abstract

The present study is aimed to investigate the antioxidative potential and ameliorative effects of *Lens culinaris* Medikus sprouts hydroalcoholic extract (LSHE) on CCl_4_-induced oxidative stress in rats. The research has been carried out in two successive stages. Firstly, the highest phenolic content and antioxidant activity of *L. culinaris* sprouts were assessed at 20 ± 1°C and 90–93% RH during sprouting. Total phenolic content (TPC), total carotenoids (TC), total flavonoids (TF), total flavonols (TFL), DPPH-RSA, and vitamin C contents of *L. culinaris* seeds and 6-days sprouts were determined. Subsequently, phenolics by HPLC analysis of *L. culinaris* seeds, 3rd and 6th-day sprouts were identified and quantified. Results indicated that 6th-day sprouts contained considerable phenolics with superior antioxidant capacity, thus selected to be examined for biological activity in a rat's module consisting of five groups. G1, normal rats orally received distilled water. G2 received 1.0 mL kg^−1^ of CCl_4_ and olive oil (1:1) intraperitoneally (i.p.) twice a week. G3 received CCl_4_ (i.p.) and 50 mg GAE kg^−1^ of LSHE daily/orally. G4 received CCl_4_ (i.p.) 100 mg kg^−1^ of LSHE orally/daily. G5 (reference group) treated by intramuscular injection (i.m.) of vit. E+Selenium (Vit. E+Se, 50 mg kg^−1^ twice a week). The weight gain, relative weight of organs, hypoglycemic and hypolipidemic efficiencies, liver's and kidneys' functions, and antioxidant biomarkers were examined. LSHE enhanced the weight gain recovery % and significantly reduced fasting blood glucose. The hypolipidemic effect of LSHE was dramatically reduced triglycerides (TG), total cholesterol (CHO), high- and low-density lipoproteins (HDL-c and LDL-c), and very-low-density lipoproteins (VLDL-c). Administration of 50 and 100 LSHE mg kg^−1^ ameliorated liver and kidney function in dose-dependent manure. Intriguingly, LSHE considerably reduced malondialdehyde (MDA) while significantly raising reduced glutathione (GSH), superoxide dismutase (SOD), and catalase (CAT) in a dose-dependent manner. In conclusion, biochemical examinations confirmed the therapeutic efficacy of LSHE as a functional product. It encouraged us to recommend *L. culinaris* sprout production for attenuating hepatotoxicity and nephrotoxicity, as well as being beneficial and profitable for controlling oxidative stress complications.

## Introduction

An imbalance among the production of reactive oxygen species (ROS) and the body's detoxification or repair mechanisms is known as oxidative stress. When the cell's redox state is disturbed, toxic peroxides and free radicals are produced, harming lipids and DNA (1). DNA strands can be broken by oxidative stress, which causes underlying damage. Indirect base damage is caused by ROS, which generates the harmful free radicals. Because some reactive oxidative species serve as cellular messengers in redox signaling, oxidative stress can interfere with cellular communication. Oxidative stress is viewed as a potential factor in the development of cancer and Alzheimer's disease in humans (2), atherosclerosis (3), and depression (4). Researchers are looking for safe and effective plant-based bioactive inhibitory compounds to combat oxidative stress.

Pulses demonstrated a significant relationship between total polyphenol and tocopherol concentration and antioxidant activity (5, 6). The amount of polyphenol, carotenoid, and tocopherol bioactive in pulses varies depending on type and cultivar (5). Lentils are among the most nutrient-dense and health-beneficial foods, according to Faris et al. (6). It includes many necessary macronutrients, such as functional proteins and carbs, essential micronutrients, and bioactive phytochemicals. Recent classifications classify lentils as a preventive and therapeutic functional food (6, 7). As one of the top five superfoods, lentils (*L. culinaris*) have recently attracted more and more attention. In addition to being high in protein, they also contain significant amounts of dietary fiber, folate, iron, and potassium (8). The abilities of green lentil extracts to act as an antioxidant and as a radical scavenger were initially documented by Amarowicz et al. (9). Using HPLC-PAD and HPLC-ESI-MS methods, phenolics were also discovered in the crude extracts. Green lentils mainly included quercetin diglycoside, *trans*-*p*-coumaric acid, procyanidin dimers, catechin and epicatechin glucosides, quercetin, and epicatechin glucosides. Xu et al. (10) examined 11 samples of Lentils to determine their antioxidant capacities and four main phenolic groups, phenolic acids, anthocyanins, flavones, and flavan-3-ols—in each sample. Different cultivars of lentils showed considerable variations in their unique phenolic compounds and chemical and cellular antioxidant activities. The highest levels of total polyphenol and antioxidant activity were found in the large and small green lentils, while the split red lentil was among the cultivars of pulses with the least amount of antioxidant activity and the lowest overall polyphenol content (5). To make functional foods or nutraceuticals to enhance consumer health, food processors may use lentils with high phenolic content and antioxidant capabilities (7, 10, 11).

A remarkable variety of secondary metabolites, minerals, and bioactive substances found in lentils have shown promise in treating and preventing numerous chronic human diseases. *In vivo* and *in vivo* studies confirmed the positive correlation between their bioactive compounds, antioxidant capacity, and related health effects (5, 6, 11, 12). However, lentils' anticancer, hypoglycemic, hypocholesterolemic, and blood pressure-reducing properties and their potential to alleviate disease were examined (6, 7, 13). It has been claimed that lentil extracts have a variety of pharmacological effects both *in vivo* and *in vitro*, including antidiabetic, hypotensive, hypolipidemic, and cardioprotective effects (7). A study has also shown that the phenolic extract from lentils possesses direct ROS scavenging abilities (14, 15). According to Jung et al. (15), the phenolic extract of *L. culinaris* can serve as a possible source of nutraceuticals with hepatoprotective properties since it partially protects liver cells from oxidative stress by triggering the cellular antioxidant system. According to Goudarzi et al. (12), sodium arsenite (SA) can significantly increase oxidative stress while also depleting antioxidant reserves and blocking the actions of antioxidant enzymes. The oxidative hepatotoxicity caused by SA may be considerably reduced with red lentil extract (RLE). Its medicinal potential might be a cheap, secure, herbal antioxidative medication to treat SA toxicity.

Researchers struggle to improve the nutritional quality of lentils and ensure their abundant supply of bioactive phytochemicals that have health-promoting activity. Sprouts are a phytonutrient-rich vegetable food that is a good source of proteins, minerals, vitamins, flavonoids, polyphenols, glucosinolates, and isothiocyanates (16). Sprouting processes an applicable procedure to enhance phenolic content, antioxidant capacity, glycemic index, and potential bioaccessibility (17). Swieca et al. (18) confirmed that sprouting improved the nutraceutical value of lentil sprouts regarding their antioxidant potential. Interestingly, according to Reed et al. (19), sprouts are regarded as “functional foods,” defined as foods with additional health-promoting or disease-prevention benefits to their primary nutritional value. Studies on sprouts' nutritional value, phytochemical makeup during production or storage, and investigations into their microbiological, bioactive, and technological aspects have been suggested (20). The nutritional advantages and sensory acceptance of food products created with other sprouts were recently reviewed in this context (21).

Until now, nobody has looked into the bio-changes in phytochemicals during lentil sprouting. Therefore, the TPC, AOA, TC, TF, TFL, and vitamin C, as well as HPLC analysis of phenolic compounds during sprouting, were studied first. Consequently, a hydroalcoholic extract of 6-days green lentil sprouts (LSHE) was prepared.

Secondly, the antioxidative potential, hepatoprotective, and nephroprotective efficacy of green lentil sprouts hydroalcoholic extract (LSHE) on CCl_4_-induced oxidative stress were investigated.

## Materials and methods

### Raw lentil seeds

Green lentil seeds (*L. culinaris* Medikus) were purchased from the Al-Tamimi market (https://www.tamimimarkets.com) in Al Qassim region, K.S.A. Plant expert (Dr. Mokded Rabhi) from the College of Agriculture and Veterinary Medicine at Qassim University in K.S.A., carried out the plant's verification. The broken, sick, and dusty seeds were eliminated. Green lentil sprouts were made from cleaned seeds. Until they were used for analysis, raw or milled seeds (American model laboratory mill, model ES2097) were kept in freezer-plastic bags at a temperature of 18 ± 1°C.

### Sprouting of *L. culinaris* and hydroalcoholic extract preparation

The seeds were sanitized into a solution of sodium hypochlorite (1%) for 3 min before sprouting in batches of 500 g. The seeds were evenly distributed on 7 × 35 cm plastic trays after being rinsed three times in sterilized distilled water (sd.H_2_O). The seed germinator was then filled with the seeds. The germination procedure was done in an atomizer-equipped temperature-controlled seed germinator (Easygreen, Canada) with a relative humidity of 90–93% with faith light. The temperature of the germinator was kept at 20 ± 1°C. For the first 3 days, 10.0 mL of sd.H_2_O/tray was sprayed onto lentil samples thrice daily. Beginning at the germination process and continuing for up to 6 days, appropriate samples were taken daily. Lentil sprouts were frozen overnight at −18 ± 1°C, then freeze-dried (CHRIST, Alpha 1-2 LD plus, Germany) for 96 h at −48°C under the pressure of 0.032 mbar. Freeze-dried sprouts were crumbled in a small mill (Thomas Wiley, USA) to obtain a homogenous powder, then kept in the dark containers at 4 ± 1°C for HPLC and phytochemical analysis. Lentil sprouts were individually germinated in identical settings for 6 days. Progressively, Lentil sprouts were dried using a 24-hdrying program according to Barakat et al. (22) and Al-Qabba et al. (23). The dried sprouts were milled, sieved, and kept under cold storage until extraction. Lentil sprouts hydroalcoholic extract (LSHE) was carried out by extracting about 500 g of lentil sprouts three times with 2,500 mL of 50% ethanol. The filtered extract was concentrated in a rotary evaporator at 40°C, then frozen overnight and freeze-dried (CHRIST, Alpha 1–2 LD plus, Germany) (24). Freeze-dried samples were powdered by porcelain morsel to make homogeneous powder which was kept under cooling conditions until used.

### Determination of TPC, TC, TF, and TFL in *L. culinaris* seeds and sprouts

According to Yawadio Nsimba et al. (25), the TPC of *L. culinaris* seeds and sprouts was determined using the Folin-Ciocalteu reagent. In brief, a suitable sample was extracted with 70% methanol. Aliquots of clear supernatant were mixed with (1:10) diluted Folin-Ciocalteu reagent for 5 min before being stopped with Na_2_CO_3_ (7.5 %). The optical density (OD) was measured after 60 min and compared to the standard curve of Gallic acid (GA) solution (*R*^2^ = 0.99), and the TPC content was expressed as milligrams of Gallic acid equivalents (GAE) per 100 g (mg of GAE 100 g^−1^ DW). For TC determination, 1 g of the freeze-dried sample was repeatedly extracted with a mixture of acetone and petroleum ether (1:1, v/v), according to Yuan et al. (26). The upper phase was collected, washed with water several times, and combined with crude extracts. The petroleum ether will be added to the solution to prepare a known volume. The TC content was determined spectrophotometrically at 451 nm and expressed as mg 100 g^−1^ dw. The upper phase was collected, washed with water several times, and combined with crude extracts. The petroleum ether will be added to the solution to prepare a known volume. The TC content was determined spectrophotometrically at 451 nm and expressed as mg 100 g^−1^ dw. The TF content of *L. culinaris* seeds and sprouts using a methanolic extract was determined. Aliquots of clear extract were mixed with 2% AlCl_3_, then measured after 60 min at 420 nm. The TFL content of *L. culinaris* seeds and sprouts was determined by combining aliquots of methanolic extracts with sodium acetate (5 %). After 5 min, AlCl_3_ (2%) was added, and the OD was measured after 150 min at 440 nm, according to Mohdaly et al. (27). The content of TF and TFL were expressed as mg quercetin equivalent (QE) per g^−1^ (mg QE 100 g^−1^).

### Vitamin C and antioxidant capacity determination

The vitamin C content using the 2,6-dichloro phenol indophenol titrimetric method was determined according to Silva et al. (28); data were expressed as mg 100 g^−1^ fw. The radical scavenging activity (DPPH-RSA) of *L. culinaris* seeds and sprouts was examined spectrophotometrically according to Barakat and Rohn (29), and antiradical activity value was presented as μmol TE 100 g^−1^.

### Quantification of phenolic compounds in *L. culinaris* and its sprouts by HPLC-DAD

According to Kim et al. (30), using an HPLC system HP1100 (Agilent Technologies, Palo Alto, CA, USA) equipped with an autosampler, quaternary pump, and diode array detector DAD, as well as an Altima C18, 5 × 150 mm, 4.6 mm ID column and an Altima C18, 5 mm guard column (Alltech), the phenolic compounds in *L. culinaris* and its sprouts were determined. At a flow rate of 1 mL min-1, 10 μl of the extracted samples were injected, and separation was carried out at 25°C. The gradient of acetic acid concentrations in the solvent system was A (acetic acid 2.5%), B (acetic acid 8%), and C (Acetonitrile 100%). For identification and quantification, each peak's retention times and mass spectra were compared to external standards and stored; then, phenolic compounds were expressed in mg Kg^−1^.

### Animals and experimental design

This study used Wistar rats (40 adult males) weighing 175–200 g. All the experiments received approval from the Institutional Animal Ethics Committee (IAEC) of QU, KSA (No. 21-18-09 on Thursday, May 19, 2022), Qassim University, SA. Under typical laboratory conditions, animals were housed in polypropylene cages with air conditioning and kept at 24°C. Rats were placed in new cages under controlled circumstances of 24°C, 40–45% relative humidity, and a 12-h light/dark cycle after being exposed to the environment for 10 days. Randomly, five groups of eight rats each were assigned to the groups. The rats' body weight (BW) was noted along with their identification labels. Rats were given a commercial standard pellet diet and unlimited access to water (31). The following procedures were used to treat the rats for six consecutive weeks. Group 1 (normal rats, NR) received 2 mL of distilled water orally/daily and an intraperitoneal injection (i.p.) of olive oil (1.0 mL kg^−1^) twice a week. For oxidative stress and hepatotoxicity induction in experimental animals. Rats were administrated (i.p.) with a fresh solution of CCl_4_ and olive oil (1:1) at a dose of 1.0 mL kg^−1^ twice a week and 2 ml of distilled water orally/daily (32). After 1 week, rats were randomly divided into four groups, eight each, and one of those groups was labeled as Group 2 and located and positive control. Group 3 received CCl_4_ (i.p.) twice a week in addition to 50 mg GAE kg^−1^ of LSHE given orally daily. Group 4 received CCl_4_ (i.p.) twice a week in addition to 100 mg kg^−1^ of LSHE administered orally daily. According to Asuku et al. (33) and Gaber et al. (34), Group 5 (reference group) received an intramuscular injection (i.m.) of Vit. E+Se (Selepherol, Vetoquinol Co., France) at 50 mg kg^−1^ twice a week and 2 mL distilled water orally daily. At the end of the 6th week, animals fasted for 12 h with free access to water. According to Leila et al. (35), rats were anesthetized. Blood was collected from the heart puncture and then treated to separate blood serum by centrifugation at 4,000 x g for 30 min for use in various biochemical measures. Appropriate kits and a blood chemistry analyzer (HumaLyzer 4000, Germany) were used to determine the biochemical parameters. Rats' liver, kidneys, and spleen were removed during the dissection of sacrificed animals. The following equation was used to determine the relative weight (RW) of the organs:


(1) $$\begin{array}{l} \text{RW }=\frac{\text{Weight of the organ}}{\text{Weight of the rat}}\times 100 \end{array}$$

### Determination of liver and kidney functions, lipid profile, and fasting blood glucose level

Alanine aminotransferase (ALT, UL^−1^), aspartate aminotransferase (AST, UL^−1^), alkaline phosphatase (ALP, UL^−1^), and total bilirubin (T. Bili, mg dL^−1^) in blood serum were measured using specific and approved kits following the manufacturing instructions. According to the manufacturer's instructions, kidney functions such as albumin (g dL^−1^), total protein (T. Protein, g dL^−1^), urea (mg dL^−1^), and creatinine (mg dL^−1^) concentrations were measured. Albumin concentrations were subtracted from T. Protein concentrations to calculate globulin (g dL^−1^). Dividing the urea concentration by 0.47, blood urea nitrogen (BUN, mg dL^−1^) was calculated. All biochemical test kits were bought from Human Co. in Wiesbaden, Germany. According to Nwagha et al. (36), the atherogenic index (AI) was calculated. An enzymatic colorimetric test kit was used to determine fasting blood glucose (mg dL^−1^). High-density lipoproteins (HDL-c, mg dL^−1^) and total cholesterol (CHO, mg dL^−1^), and triglycerides (TG, mg dL^−1^), according to manufacturer instructions, were examined. According to Friedewald et al. (37), low-density lipoproteins (LDL, mg dL^−1^) and very-low-density lipoproteins (VLDL, mg dL^−1^) were mathematically calculated.

### Oxidative stress biomarkers

According to the described method by Beutler et al. (38), reduced-glutathione (GSH, μg dL^−1^) was determined. According to Ohkawa et al. (39), lipid peroxidation was evaluated by detecting thiobarbituric acid reactive substance (TBARS), and the measured malondialdehyde (MDA) concentration was expressed as nmol mL^−1^. Superoxide dismutase (SOD, U L^−1^) activity was determined following the protocol of Giannopolitis and Ries (40). Catalase (CAT, U L-1) activity was assessed using the Aebi technique (41). All oxidative-stress biomarkers were determined using a blood chemistry analyzer (HumaLyzer 4000, Germany).

### Data analysis

The SPSS (Ver. 22.0 for Windows) was used to conduct the statistical analysis. According to Steel et al. (42), one-way ANOVA was used to assess the statistical significance, *p*-values of 0.05 were used for the *post-hoc* test, and means of the experimental results and standard error were presented.

## Results

### The phytochemicals and antioxidant activity of *L. culinaris* sprouts

The quantitative analysis of phytochemicals such as TPC, TC, TF, and TFL, as well as related antioxidant activity using DPPH radical scavenging and vitamin C content in *L. culinaris* sprouts, was performed. The TPC content of green lentil seeds (GLS) was 379.76 mg GAE 100 g^−1^, as illustrated in Table 1. The TC content of GLS was 14.15 g 100 g^−1^. Both TF and TFL contents in GLS were 16.32 and 11.17 mg QE 100 g^−1^, respectively. Furthermore, the development of antioxidant activity was tracked using DPPH-RSA. The results showed 479.42 mol of TE 100 g^−1^ in GLS. The vitamin C content of GLS was 42.91 mg 100 g^−1^. On the 6th day, significant increases in TPC, TC, TF, and TFL, as well as Vit. C were observed.

**Table 1:** Total phenolic, carotenoids, flavonoids, flavonols contents, and potential antioxidant capacities of *L. culinaris* during sprouting at 20 ± 1°C and 90–93% RH (mean ± SE), *n* = 6.

**Item**	**Sprouting period (day)**						
	**0**	**1**	**2**	**3**	**4**	**5**	**6**
TPC (mg meq GAE 100 g^−1^)	379.76 ± 14.15^f^	461.78 ± 20.82^e^	557.55 ± 11.89^d^	620.45 ± 14.66^c^	678.89 ± 17.85^b^	770.87 ± 6.36^a^	788.78 ± 5.26^a^
TC (μg 100 g^−1^)	13.00 ± 0.83^d^	10.89 ± 0.28^d^	16.47 ± 0.88^c^	19.71 ± 0.20^b^	21.15 ± 0.63^b^	24.48 ± 1.16^a^	25.17 ± 1.80^a^
TF (mg QE 100 g^−1^)	16.32 ± 4.38^d^	20.43 ± 6.45^cd^	39.47 ± 7.42^bc^	52.91 ± 4.31^ab^	56.88 ± 9.50^ab^	64.89 ± 10.93^a^	52.40 ± 5.42^ab^
TFL (mg QE 100 g^−1^)	11.17 ± 3.64^b^	14.41 ± 4.50^b^	17.40 ± 1.62^b^	36.28 ± 6.75^a^	38.30 ± 3.65^a^	44.42 ± 5.85^a^	45.59 ± 2.29^a^
DPPH (μmol of TE 100 g^−1^)	479.42 ± 25.39^c^	515.19 ± 27.58^bc^	547.04 ± 37.76^bc^	565.01 ± 22.14^bc^	594.24 ± 35.74^bc^	679.57 ± 31.31^a^	539.73 ± 45.21^ab^
Vitamin C (mg 100 g^−1^)	42.91 ± 1.10^e^	40.76 ± 1.04^e^	60.07 ± 1.54^d^	72.94 ± 1.87^c^	92.25 ± 2.36^b^	93.75 ± 2.40^b^	122.54 ± 3.14^a^

TPC, Total phenolic content; TC, Total carotenoids; TF, Total flavonoids; TFL, Total flavonols; DPPH, Antioxidant activity using DPPH assay; RH, Relative humidity;

a,b,c,d,e,f , Means with the same superscripted letters in the same row are not statistically different at p < 0.05.

### Quantification of phenolic compounds in *L. culinaris* seeds and sprouts

The quantitative analysis of phenolics in extracts of *L. culinaris* seeds and sprouts was carried out; the data are illustrated in Table 2. Thirteen separated phenolic acids and five flavonoids were identified in GLS and its sprouts in detectable amounts. The most abundant phenolics were *p*-Hydroxy benzoic acid (71.34 mg Kg^−1^), followed by *p*-coumaric acid (38.46 mg Kg^−1^) and Vanillic acid (21.40 mg Kg^−1^). The GLS is abundant in TF content, as demonstrated in Table 2. TF such as Naringenin (112.62 mg Kg^−1^) and Quercetin (35.29 mg Kg^−1^) were identified and found in higher amounts, followed by Myricetin (28.58 mg Kg^−1^), Resveratrol (19.00 mg Kg^−1^) and Kaempferol (15.27 mg Kg^−1^). On the 3rd day of sprouting, Benzoic acid, Rosmarinic acid, and Syringic acid as phenolic acids were presented in 42.73, 28.48, and 28.48 mg Kg^−1^, respectively. Similarly, new flavonoids have been shown in reasonable amounts with an increase in detected flavonoids in GLS except for Naringenin. Rutin and Catechin were quantified at 50.02 and 4.19 mg Kg^−1^ in the 3rd day's sprouts. Seven flavonoids, eleven phenolic acids, and their derivatives were identified and measured on the 6-day. The most abundant phenolic acids were Rosmarinic acid, *p*-Hydroxy benzoic acid, and *p*-coumaric acid. In contrast, the most abundant flavonoids were Kaempferol, followed by Myricetin, Naringenin, Resveratrol, Quercetin, Rutin, and Catechin. On the 6th day, phenolic acids and flavonoids were remarkably increased, whereas the flavonoids recorded increases higher than phenolic acids and their derivatives.

**Table 2:** Quantitative analysis of phenolic compounds in *L. culinaris* during sprouting at 20 ± 1 °C and 90–93% RH (mean ± SE), *n* = 3.

**Item**	**No**.	**Compound**	**Phenolics (mg Kg**^**−1**^**)** ^*^		
			**Sprouting period (day)**		
			**0**	**3**	**6**
Phenolic acids	1	Pyrogallol		–	
	2	Quinol	–	–	–
	3	3–Hydroxytyrosol catechol	–	–	–
	4	*p*–Hydroxy benzoic acid	71.34 ± 3.24	82.05 ± 1.10	73.93 ± 2.57
	5	Caffeic acid	1.25 ± 0.24	2.70 ± 0.87	3.34 ± 1.24
	6	Chlorogenic acid	2.16 ± 0.29	2.56 ± 0.19	4.19 ± 0.54
	7	Cinnamic acid	0.69 ± 0.12	0.41 ± 0.21	0.28 ± 0.08
	8	Ellagic acid	–	–	–
	9	Vanillic acid	21.40 ± 0.21	0.91 ± 0.14	59.20 ± 4.25
	10	Ferulic acid	6.45 ± 1.02	2.96 ± 0.85	10.32 ± 1.98
	11	Gallic acid	–	–	–
	12	*O* – coumaric acid	7.61 ± 0.97	6.33 ± 0.71	5.14 ± 0.26
	13	*p*–coumaric acid	38.46 ± 2.18	76.61 ± 3.48	59.75 ± 6.18
	14	Benzoic acid	–	42.73 ± 2.98	61.48 ± 2.78
	15	Rosmarinic acid	–	28.48 ± 1.97	114.88 ± 5.19
	16	Syringic acid	–	3.83 ± 0.58	5.19 ± 0.79
Flavonoids	1	Catechin	–	4.19 ± 0.25	14.90 ± 2.97
	2	Kaempferol	15.27 ± 1.57	166.86 ± 6.27	4439.54 ± 10.24
	3	Myricetin	28.58 ± 1.25	134.72 ± 4.97	224.16 ± 5.27
	4	Quercetin	35.29 ± 2.21	41.89 ± 3.19	54.12 ± 5.27
	5	Rutin	–	50.02 ± 4.87	39.01 ± 5.02
	6	Resveratrol	19.00 ± 2.75	54.62 ± 2.97	80.64 ± 3.97
	7	Naringenin	112.62 ± 4.21	89.78 ± 6.12	142.57 ± 8.02

* : Phenolic acids were identified at 280 nm, and flavonoids were identified at 365 nm, Not detected.

### The weight gain, the RW of organs, and hypoglycemic efficiency

The weight gain, RW of organs, and hypoglycemic efficiency of GLR extracts in CCl_4_-induced oxidative stress and hepatotoxicity in rats were scrutinized; data are presented in Table 3. Injection of CCl4 directly affected the rats' weight during the 1st week; however, virtually little weight gain was observed in G2 rats in the 6th week. The most efficient treatment in recovering rats' weight was administering 100 mg GAE Kg^−1^ LSHE compared with G1 or G5 at the end of the 6th week. LSHE significantly improved weight gain associatively in a dose-dependent manner. For organs' relative weight, the injected group exhibited significant increases in organs' weight. Treating rats with LSHE or Vit. E+Se showed a positive attenuation. After the 6th week, LSHE at 50 or 100 mg GAE Kg^−1^ exhibited a potent efficacy in reducing fasting blood glucose but not better Vit. E+Se at 50 mg Kg^−1^, as shown in Table 3.

**Table 3:** Effect of *L. culinaris* sprouts hydroalcoholic extract on weight gain%, organs' weight, and FBG in CCl_4_-induced oxidative stress and hepatotoxicity in rats (mean ± SE), *n* = 8.

**Groups**	**Weight gain %**		**Organs' relative weight (%)**			**FBG**
	**3rd weak**	**6th weak**	**Liver**	**Kidneys**	**Spleen**	
G1	31.41 ± 2.35^a^	44.23 ± 3.27^a^	3.24 ± 0.02^b^	0.79 ± 0.04^a^	0.39 ± 0.02^a^	78.43 ± 4.63^b^
G2	−0.33 ± 0.24^d^	3.63 ± 1.25^d^	3.71 ± 0.10^a^	0.84 ± 0.02^a^	0.42 ± 0.01^a^	133.02 ± 7.34^a^
G3	16.08 ± 3.24^c^	22.64 ± 2.48^c^	3.24 ± 0.02^b^	0.67 ± 0.02^b^	0.33 ± 0.01^b^	106.82 ± 6.00^b^
G4	23.67 ± 2.87^b^	36.78 ± 4.28^b^	2.91 ± 0.15^b^	0.66 ± 0.03^b^	0.33 ± 0.01^b^	94.25 ± 5.38^b^
G5	26.59 ± 1.49^b^	36.82 ± 3.98^b^	3.15 ± 0.10^b^	0.78 ± 0.01^a^	0.39 ± 0.01^a^	84.49 ± 5.49^b^

G1–G5, Experimental groups see materials and methods; section 2.6, FBG, Fasting blood glucose,

a,b,c,d : Means with the same superscripted letters in the same column are not statistically different at p < 0.05.

### The hypolipidemic efficiency

The hypolipidemic efficiency of LSHE at 50 and 100 mg GAE Kg^−1^ and Vit. E+Se at 50 mg kg^−1^ on CCl_4_-induced oxidative stress and hepatotoxicity in rats were determined; data are shown in Table 4. The CCl_4_-induced oxidative stress and hepatotoxicity in rats were observed to significantly raise the levels of TG, CHO, LDL-c, and VLDL-c. However, CCl_4_ injection resulted in significantly lower HDL-c levels than in control rats (G1). The lipid profile was improved in dose-dependent manure by administering LSHE at 50 or 100 mg GAE Kg^−1^. The most effective therapy for enhancing the blood profile was LSHE with 100 mg GAE Kg^−1^, which showed no significance when compared to G1 or G5. However, comparing G5 (41.55%), administrating LSHE at 50 or 100 mg GAE Kg^−1^ reduced the TG level by 33.56 and 34.44%, respectively. Interestingly, the rate of CHO reduction was 33.11, 39.55, and 41.61% for treating rats with 50, 100 mg GAE Kg^−1^ of LSHE and 50 mg Kg^−1^ Vit. E+Se, respectively. HDL-c increase was recorded as 12.92, 41.95, and 34.78%, whereas LDL-c decrease was noted as 47.12, 65.37, and 64.64% after LSHE at 50, 100 mg GAE Kg^−1^ or 50 mg Kg^−1^ Vit. E+Se treatments, respectively. VLDL-c level was improved with treatments associatively in a type and dose-dependent manner. LSHE with 100 mg LSHE Kg^−1^ was significantly better than 50 mg LSHE Kg^−1^. Fascinatingly, when CCl_4_ was injected, the AI significantly raised compared to normal rats (G1). Indeed the most efficient treatments for attenuating the atherogenicity complication were those giving 100 mg GAE Kg^−1^ of LSHE, which presented better attenuation than 50 mg GAE Kg^−1^ of LSHE and even normal rats. The superior effect was recorded for using 50 mg Kg^−1^ Vit. E+Se did not differ significantly from using 100 mg GAE Kg^−1^ of LSHE.

**Table 4:** Effect of hydroalcoholic extract of *L. culinaris* sprouts on lipid profile and Atherogenic index in CCl_4_-induced oxidative stress and hepatotoxicity in rats (mean ± SE), *n* = 8.

**Groups**	**Lipid profile parameters**					
	**TG**	**CHO**	**HDL-c**	**LDL-c**	**VLDL-c**	**AI**
G1	85.5± 1.80^bc^	102.30± 10.34^b^	39.29± 02.80^bc^	45.92± 11.67^b^	17.10± 0.36^bc^	0.34± 0.02^b^
G2	137.19± 4.4^a^	170.87± 7.71^a^	33.21± 2.58^b^	110.22± 8.51^a^	27.44± 0.88^a^	0.62± 0.04^a^
G3	92.52± 1.94^b^	114.29± 4.61^b^	37.50± 0.82^b^	58.29± 4.37^b^	18.51± 0.39^b^	0.39± 0.03^b^
G4	89.94± 3.28b^c^	103.29± 5.74^b^	47.14± 1.05^a^	38.16± 5.42^b^	17.99± 0.66^bc^	0.28± 0.01^c^
G5	80.19± 2.26^c^	99.78± 4.47^b^	44.76± 2.03^a^	38.97± 5.45^b^	16.04± 0.45^c^	0.25± 0.05^c^

G1–G5, Experimental groups see materials and methods; TG, Triglycerides; CHO, total cholesterols; HDL-c, High-density lipoprotein-cholesterols; LDL-c, Low-density lipoprotein-cholesterols; VLDL-c, Very low-density lipoprotein-cholesterols; AI, Atherogenic index,

a,b,c : Means with the same superscripted letters in the same column are not statistically different at p < 0.05.

### The liver's functions

**Figure 1:**
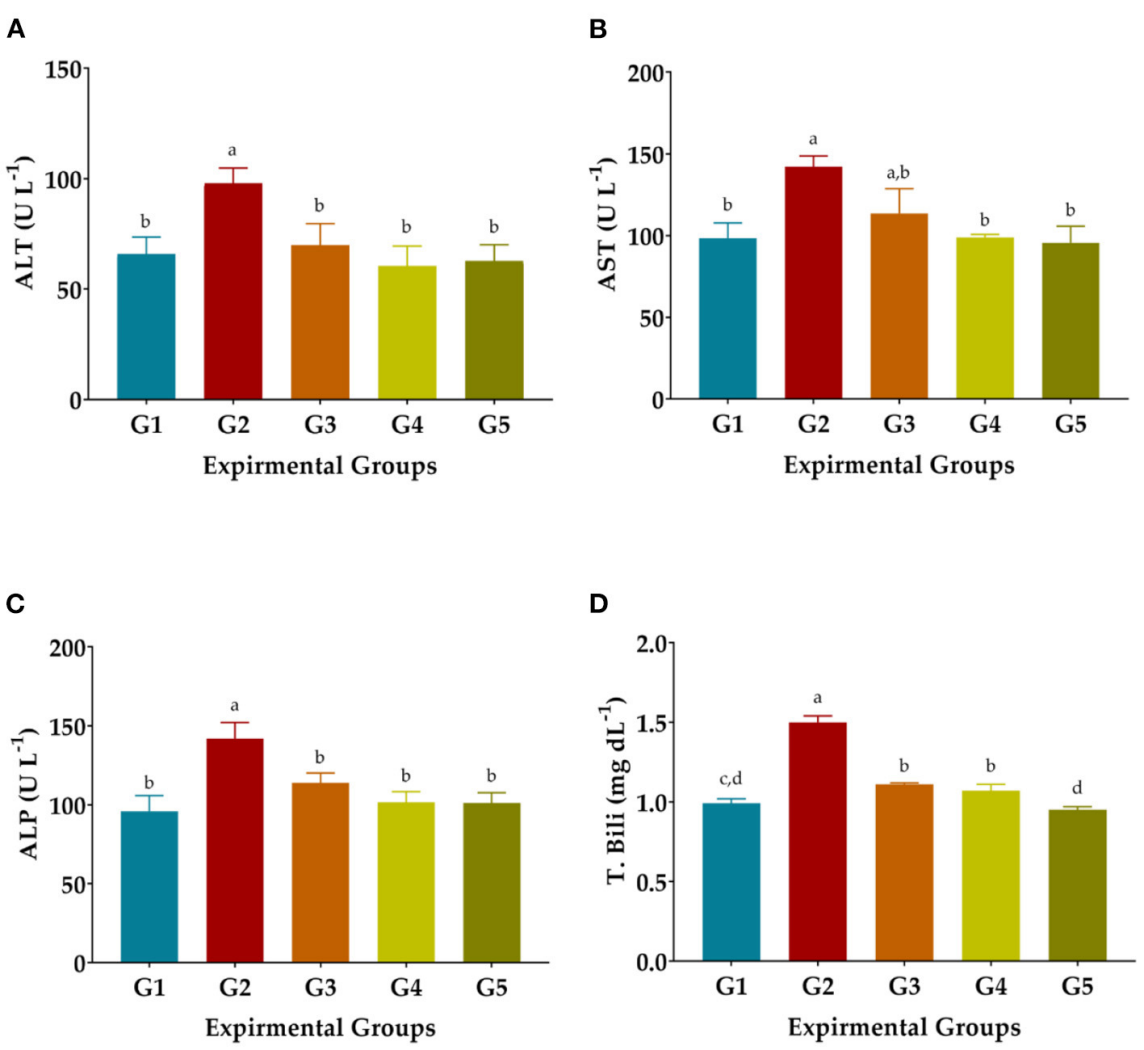
Effect of hydroalcoholic extract of *L. culinaris* sprouts on liver's functions in CCl_4_-induced oxidative stress and hepatotoxicity in rats (mean ± SE), *n* = 8. **(A)** ALT: Alanine aminotransferase. **(B)** AST, Aspartate Aminotransferase. **(C)** ALP, Alkaline phosphatase. **(D)** T. Bili, Total bilirubin, ^a,b,c,d^: Bars not sharing similar letters are statistically different at *p* < 0.05.

CCl_4_ injection considerably raised serum AST, ALT, and ALP enzyme levels in G2 rats as oxidative stress and hepatotoxicity complications compared to normal rats (GI). The T. Bili level was significantly increased in CCl_4_-treated rats (Figure 1). Administration of LSHE at 50 or 100 mg GAE Kg^−1^ and Vit. E+Se at 50 mg Kg^−1^ improved the liver's function. A high level of LSHE was better than a low level of LSHE or Vit. E+Se to improve liver functions. Interestingly, giving LSHE reduced the modifications in liver functions caused by CCl_4_ injection to be close to typical values in GI (Figure 1). The ALT level attenuated by 28.68, 38.22, and 35.94% when 50 and 100 mg GAE LSHE Kg^−1^ and 50 Kg^−1^ Vit. E+Se were given, respectively. Similarly, ATS and ALP improved by 20.01, 30.44, and 32.71% and 19.83, 28.48, and 28.80%, respectively. However, in comparison to NR in G1, LSHE, and Vit. E+Se significantly enhanced some liver functions such as T. Bili and the liver enzymes as shown in (ALT, ALP, and AST). T. Bili level was attenuated by 26.00, 28.67, and 36.67% when rats were administrated when 50 and 100 mg GAE LSHE Kg^−1^ and 50 Kg^−1^ Vit. E+Se, respectively.

### The kidneys' functions

The nephroprotective efficiency of LSHE at 50 or 100 mg GAE Kg^−1^ and Vit. E+Se at 50 mg Kg^−1^ on CCl_4_-induced oxidative stress and hepatotoxicity in rats were studied; results are demonstrated in Table 5. CCl_4_ injection considerably increased serum urea, BUN, and creatinine levels in G2 rats compared to NR in GI. Conversely, albumin, T. Protein, and globulin levels were drastically decreased in CCl_4_-treated rats (Table 5). LSHE at 50 or 100 mg GAE Kg^−1^ and Vit. E+Se at 50 mg Kg^−1^ treatments significantly attenuated urea, creatinine, and BUN alterations caused by CCl_4_ problems. Albumin, T. Protein, and Globulin levels were also raised to nearly normal levels in the GI (Table 5). The most effective enhancement was evidently recorded with LSHE at 100 mg GAE Kg^−1^ even sometimes better than using Vit. E+Se at 50 mg Kg^−1^ compared to normal rats (G1).

**Table 5:** Effect of hydroalcoholic extract of *L. culinaris* sprouts on kidneys' functions in CCl_4_-induced oxidative stress and hepatotoxicity in rats (mean ± SE), *n* = 8.

**Group**	**Kidneys' functions**					
	**T. Protein (g dL^−1^)**	**Albumin (g dL^−1^)**	**Globulin (g dL^−1^)**	**Creatinine (mg dL^−1^)**	**Urea (mg dL^−1^)**	**BUN (mg dL^−1^)**
G1	9.80± 1.00^a^	4.59± 0.23^a^	5.21± 0.86^a^	0.89± 0.09^b^	86.96± 8.03^bc^	40.87± 3.77^bc^
G2	7.44± 0.21^a^	3.08± 0.27^b^	4.36± 0.37^a^	1.47± 0.22^a^	156.52± 5.01^a^	73.57± 2.36^a^
G3	8.72± 0.43^a^	3.90± 0.37^ab^	4.82± 0.71^a^	0.70± 0.04^b^	105.31± 8.52^b^	49.50± 4.01^b^
G4	9.51± 0.61^a^	4.49± 0.62^ab^	5.02± 0.69^a^	0.76± 0.02^b^	90.10± 10.77^b^	42.35± 5.06^b^
G5	9.13± 0.87^a^	4.69± 0.34^a^	4.44± 0.88^a^	0.81± 0.03^b^	80.96± 3.19^c^	38.05 ± 1.50 ^b^

G1–G5, Experimental groups see materials and methods, BUN; Blood urea nitrogen,

a,b,c , Means with the same superscripted letters in the same column are not statistically different at p < 0.05.

### Antioxidant biomarkers

**Figure 2:**
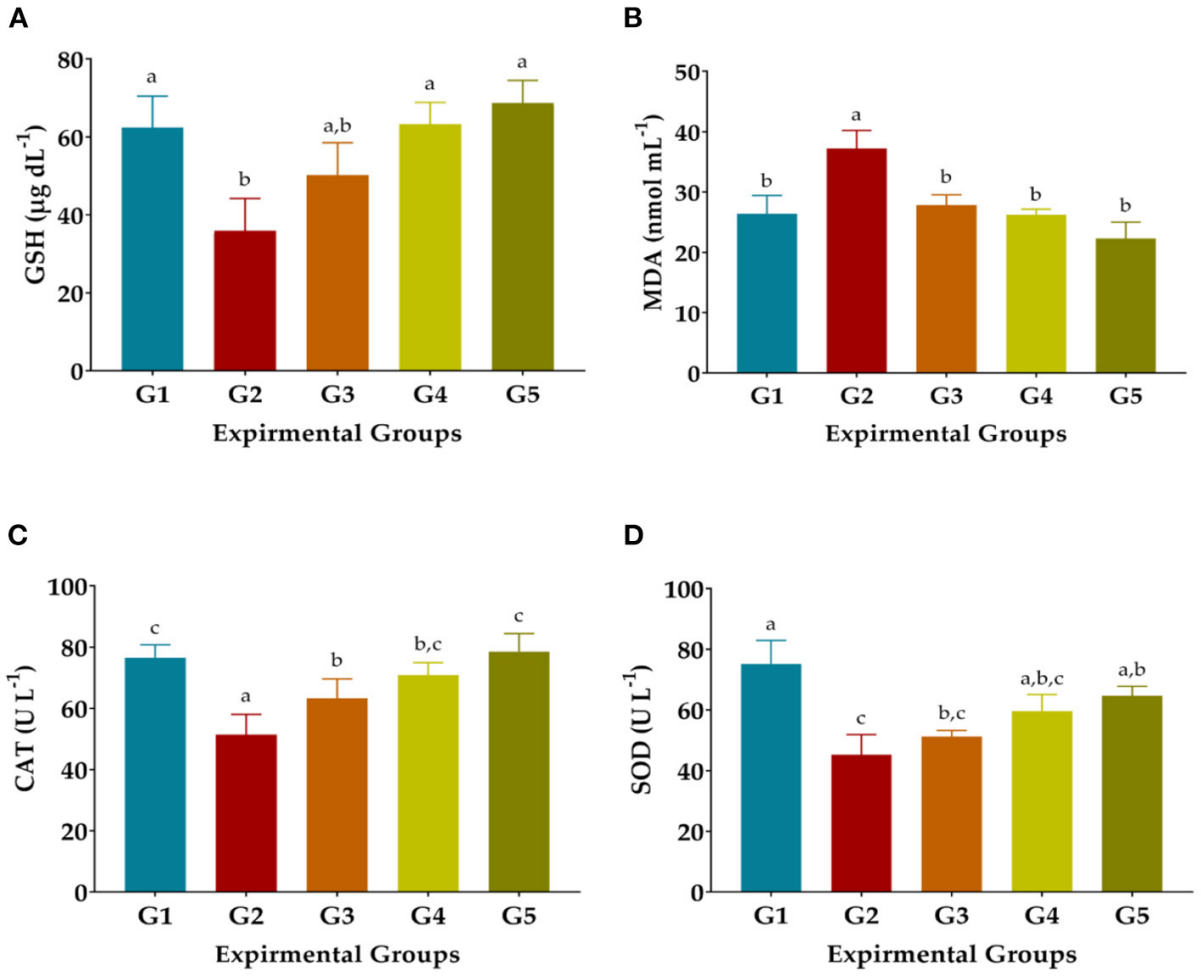
Effects of oral administration of hydroalcoholic extract of *L. culinaris* sprouts on antioxidant biomarkers in CCl_4_-induced oxidative stress and hepatotoxicity in rats (mean ± SE), *n* = 8. **(A)** GSH: Reduced glutathione, **(B)** Malondialdehyde, **(C)** CAT: Catalase, and **(D)** SOD: Superoxide dismutase. G1–G5: Experimental groups see materials and methods, ^a,b,c,d^: Bars not sharing similar letters are statistically different at *p* < 0.05.

Injection of CCl4 dramatically decreased GSH, SOD, and CAT levels and elevated MDA levels in the blood serum of G2 compared to NR in G1, as shown in Figure 2. The activity of the antioxidant enzymes GSH, CAT, and SOD was significantly improved in the treated rats treated with 50 or 100 mg GAE Kg^−1^ and Vit. E+Se at 50 mg Kg^−1^ and the levels of MDA were significantly decreased, as shown in Figure 2. On the other hand, treatment of 50 mg LSHE Kg^−1^ exuded minimal diminution in GSH, CAT, and SOD and inhibited the autoxidation process, resulting in low MDA levels. Compared to the CCl_4_-group (G2), the most effective treatment for GSH, DMA, CAT, and SOD was LSHE with 100 mg Kg^−1^, which showed improved rates of 76.36, 29.47, 37.82, and 31.72 %, respectively. Comparing rats treated with CCl_4_ and normal rats (G1), it is interesting to note that rats treated with 50 mg Kg^−1^ of vit. E+Se dramatically improved their enzymatic defense system (G2).

## Discussion

Functional foods regulate diabetes through blood pressure regulation, activation of antioxidant enzymes, interaction with gut microbiota, suppressing of pro-inflammatory cytokine overproduction, and presenting antioxidative potential (23, 43, 44). A capable strategy, particularly when phenolics are incorporated, has been described (32) with superior antioxidant activity (45). The valuable phytochemical content and antioxidant activities of LSHE corresponded to Amarowicz et al. and Xu and Chang (9, 10). Indeed, biologically active components, such as phenolic acids and flavonoids, demonstrate antioxidant capacity by stopping lipid oxidation chain reactions *in vitro* and *in vivo* (9, 10). The phenolics' ability to scavenge and inhibit free radicals is caused by the phenolic hydroxyl groups located in polyphenols (46, 47). An efficient antioxidant component has been presented in various phenolic acids inhibiting hydrogen peroxide formation, superoxide anion, and hydroxyl radicals (48, 49). A direct correlation exists between abundant polyphenols concentration and their antioxidant function (9, 10, 23, 50).

Consumption of sprout extracts could help reduce cellular oxidation, as confirmed in the current study (23, 51). Interestingly during sprouting, phenolics and antioxidants increased (18). Zhang et al. (52) evaluated the total phenolic composition and contents, antioxidant activities (DPPH, FRAP, ORAC), and inhibitory properties of phenolic extracts from 20 Canadian lentil cultivars (*L. culinaris*) against α-glucosidase and pancreatic lipase (52). All extracts showed antioxidant and radical-scavenging properties as revealed by the total antioxidant activity (TAA) method, a β-carotene-linoleate model system, a reducing power assay, and the DPPH scavenging activity assay (9).

The increased number of phenolics in *L. culinaris* sprouts increased than its seeds with the progression of the sprouting period; results were agreed with Swieca and Gawlik-Dziki (18). Remarkably, present research noticed a considerable amount of identified phenolics higher than confirmed previously (9). However, Amarowicz et al. (9) indicated increased Rosmarinic acid, *p*-coumaric acid, and *p*-Hydroxy benzoic acid, which was consistently observed in the current study. It is worth mentioning that the phenolics profile in the cotyledon and seed coat of lentils vary and are affected by numerous factors (53, 54). *L. culinaris* seeds and sprouts show superior flavonoids content, similarly presented in 4 lentil varieties (55). Zhang et al. (52) supported our study regarding identified phenolics in *L. culinaris* seeds. In the present study, twenty-one phenolic compounds were identified, with the majority being flavonoids, including catechin/epicatechin glucosides, kaempeferol glycosides, and procyanidins. Amarowicz et al. (9). Twenty compounds (procyanidins, hydroxycinnamates, flavonols, gallates, dihydroflavonols, and dihydrochalcones) were detected and quantified in the crude extracts by HPLC-PAD and HPLC–ESI-MS procedures. The dominant phenolics in GL were epicatechin glucosides, catechin, procyanidin dimers, quercetin diglycoside, and trans-*p*-coumaric acid.

In addition to being beneficial against various metabolic illnesses, biologically active substances such as phenolic components have been defined as useful antioxidant substances, including hydroxyl radicals, hydrogen peroxide, and superoxide anion (56, 57). Interestingly, quantifying phenolics in *L. culinaris* sprouts indicated considerable numbers of phenolic acids and flavonoids, which increased significantly with increased sprouting time as confirmed (17, 18, 23) to process biological and nutritional benefits (20, 21).

Indeed, oxidative stress is thought to lead to many metabolic impairments, such as hypoglycemia (58–60). Our recent *in vivo* study indicated that LSHE stated substantial reductions in FBG in rats in a dose-dependent manner, as similarly indicated (13, 23, 51). These results strengthen our analysis, which confirms that LSHE possesses hypoglycemic effects because of rich polyphenols as effective antioxidants capable of modulating glucose levels (9, 15). Practically, the administration of LSHE was extremely beneficial in body weight recovery in a dose-dependent manner (57, 61).

Our results demonstrated the efficacy of LSHE as a rich source of antioxidants that alleviated liver malfunctions and elevated serum lipids among CCl_4_-intoxicated rat groups. It might be due to increasing and supporting rats' serum with bioactive dietary antioxidants (9, 15, 62). Administration of LSHE at 50 or 100 mg GAE Kg^−1^ improved the lipid profile in dose-dependent manure. The highly efficient therapy for improving the blood profile was LSHE with 100 mg GAE Kg^−1^ which presented no significance compared to normal rats (G1) or (G5). Obviously, administrating 50 mg Kg^−1^ Vit. E+Se showed the highest improvement rate (41.55%) in lipid profile because of high antioxidant content, as similarly observed (15, 62). Excitingly, the VLDL-c level was adjusted associatively in a dose-dependent manner with LSHE treatments. Our results align with Morise et al. (63), who explained that flaxseed oil rich in α-linolenic acid caused an elevated cholesterol secretion, causing depletion of the intrahepatic pool of cholesterol resulting in cholesterol synthesis increases. Additionally, α-linolenic acid reduced hepatic lipid accumulation by stimulating β-oxidation and suppressing fatty acid synthesis (12, 15, 64). Concerning TG, high antioxidants can be assigned to a decrease in the hepatic synthesis of fatty acids, decreasing the triacylglycerol concentration in the liver and reducing autoxidation, which attenuates VLDL-c accumulation (15, 65). Interestingly, 100 mg GAE Kg^−1^ of LSHE was more effective than 50 mg GAE Kg^−1^ of LSHE or even normal rats in attenuating the atherogenicity issue. Better results were obtained with 50 mg Kg^−1^ vit. E+Se, which did not significantly differ from 100 mg GAE Kg^−1^ of LSHE (15).

CCl_4_ insertion in a rat enlarged its liver by accumulating fats inside liver cells (66). Elevated serum enzymes activities levels (AST, ALT) signify cellular leak and loss of efficient integrity of cell membranes in the liver because of CCl_4_ intoxication. Administration of LSHE or Vit. E+Se significantly improved the levels of liver enzymes (ALT, AST) which consistently agreed (51, 62). Similarly, Saxena et al. (67) and Jung et al. (15) have confirmed the effects of the plant-based extract on elevated serum AST and ALT enzymes against oxidative stress induced by CCl_4_. T.Bili indicates that liver damage and CCl_4_ had a considerably higher level than in treated groups (Vit. E+Si and LSHE) or the NR group. Incidentally, LSHE was also efficient as Vit. E+Se presented. A current study has also indicated that in valuable amounts, LSHE contains rosmarinic acid, *p*-coumaric acid, vanillic acid, *p*-Hydroxy benzoic acid, and Benzoic acid, as well as high content of flavonoids such as kaempferol, myricetin, quercetin, resveratrol, Naringenin, and rutin. Antioxidative and anti-inflammatory efficiency in rats with hepatic damage has been proven with these compounds (68). Due to more polyphenols (20, 21), LSHE effectively attenuated oxidative stress complications. Therefore, LSHE may offer superior liver protection by blocking the development of liver fibrosis and suppressing TGF-β1 (68).

A correlation between nephrotoxicity and oxidative stress has been exhibited in many investigational models (69), and our study's results in kidney functions proved the same pattern as the organ function markers (61). Presented data clearly showed the recovery in all Kidney functions with orally administered LSHE for up to 6 weeks in dose-dependent manure. The elevated levels of Albumin, T. Protein, and Globulin and decreases in Urea, Creatinine, and BUN were highly meaningful in G4 and G5 compared to all other treated groups. An enhancement of kidney function parameters to the normal level in CCl_4_-injected rats fed *Anastatica hierochuntica* ethanolic and aqueous extracts (61) Samsum Ant *Pachycondyla sennaarensis* Venom (69) was demonstrated. Concerning the positive impact of LSHE on Kidneys' function attenuation, it was previously described that caffeic acid, carnosic acid, rosemarinic acid, and essential oil are accountable for the body's protection against free radical attack through occurred oxidative stress (6, 9, 15).

As established by the catabolite MDA indicator, tissue damage and lipid peroxidation are mediated by generated ROS (70). ROS enhances the risk of tissue injury and causes lipid peroxidation, as ascertained by the catabolite malondialdehyde marker (70). Earlier experiments indicated that CCl_4_ i.p. injection drastically decreased SOD, CAT, GPx, and GSH activities but considerably enhanced the MDA level (23, 71). Owning rich polyphenols content and AOA in LSHE, administrating 100 mg LSHE kg^−1^ was more efficient in attenuating autoxidation. The enzymatic antioxidant defense system such as SOD, CAT, and glutathione enzymes are essential scavengers of active radicals (68). LSHE attenuated GSH, CAT, SOD, and MDA levels close to the NR and equal to administrating Vit. E+Se. Previous studies have reported that consuming *L. culinaris* (6, 9, 15) increased serum antioxidant enzymes (51, 72). Concerning the present study's observations, administering LSHE orally increased the antioxidant enzymes SOD and CAT levels and decreased lipid peroxidation in CCl_4_-injected rats (57). The efficiency was significantly improved when LSHE was given at 100 mg Kg^−1^. As phenolics and antioxidants increase during sprouting (18), the consumption of sprouts extracts could help reduce cellular oxidation (23, 51). Also, LSHE attenuated the MDA and restored the total antioxidant defense in the CCl_4_-treated rats. This protective efficiency may be due to the potent antioxidative capacity of LSHE in the presence of high polyphenols, which effectively diminishes the complications related to oxidative stress (20, 21, 49, 73, 74).

## Conclusions

This study investigated and confirmed the antioxidative potential of *L. culinaris* Medikus. The current study looked into a rat module's antioxidative, hepatoprotective and nephroprotective properties of L. culinaris sprouts extract. It is possible to conclude that the *L. culinaris* sprouts extract is high in phenolic compounds, particularly flavonoids with high antioxidant capacity. Phenolic analysis revealed that *L. culinaris* sprouts contained significant amounts of TF, which support its functional and therapeutic properties. Compared to vit. E+Se administration of LSHE at 50 and 100 mg Kg^−1^ protects rats against CCl_4_ oxidative stress. The protective efficacy could be attributed to the high concentration of phenolics (e.g., rosmarinic acid, *p*-hydroxybenzoic acid, vanillic acid, *p*-coumaric acid, benzoic acid, kaempferol, naringenin, myricetin, resveratrol, and quercetin) which may modulate glucose levels and reduce hepatotoxicity complications. In addition, biochemical examinations have confirmed this superior activity. As a result, the findings could aid in explaining the therapeutic efficacy of LSHE as a functional product. It encouraged us to recommend *L. culinaris* sprout production for combining oxidative stress, as well as being beneficial and profitable for controlling oxidative stress complications.

## Data availability statement

The original contributions presented in the study are included in the article/supplementary material, further inquiries can be directed to the corresponding author/s.

## Ethics statement

The animal study was reviewed and approved by the Committee of Research Ethics, Deanship of Scientific Research, Qassim University (No. 21-18-09 on Thursday, May 19, 2022), SA, governed by the Control and Supervision of Experiments on Animals (CPCSEA) Committee of the National Committee of BioEthics (NCBE), which implements regulations related to the ethics of research on living creatures.

## Author contributions

SIA and HB: research design. ASA and RIA: experiment performance. SIA and HB: experiment operation assistance. SIA and RMA: main supervision and research leadership. RAA, RMG, and MA: draft manuscript writing. AAHA: validation and formal analysis. TA and HB: manuscript writing. All authors contributed to the article and approved the submitted version.

## Conflict of interest

The authors declare that the research was conducted in the absence of any commercial or financial relationships that could be construed as a potential conflict of interest.

## Publisher's note

All claims expressed in this article are solely those of the authors and do not necessarily represent those of their affiliated organizations, or those of the publisher, the editors and the reviewers. Any product that may be evaluated in this article, or claim that may be made by its manufacturer, is not guaranteed or endorsed by the publisher.

## Abbreviations

ALT: Alanine aminotransferase
AOA: Antioxidant activity
AST: Aspartate aminotransferase
CAT: Catalase
CHO: Total cholesterol
DPPH: 1,1-diphenyl-2-picryl hydrazine
dw: Dry weight
GA: Gallic acid
GAE: Gallic acid equivalent
GSH: Reduced-glutathione
HDL-c: High-density lipoproteins
LDL-c: Low-density lipoproteins
LSHE: *Lens culinaris* Medikus sprouts hydroalcoholic extract
MDA: Malonaldehyde
QE: Quercetin equivalent
RSA: Radical scavenging activity
SE: Standard error
SOD: Superoxide dismutase
TBA: Thiobarbituric acid
TC: Total carotenoids
TE: Trolox equivalent
TF: Total flavonoids
TFL: Total flavonols
TG: Triglycerides
TPC: Total phenolic compounds
VLDL-c: Very Low-density lipoproteins.
